# Time spent following the Russian-Ukranian war (RUW) and psychological distress: The role of sleep problems

**DOI:** 10.1177/20551029251405054

**Published:** 2025-12-07

**Authors:** Taina Hintsa, Petri Karkkola, Juhani Julkunen, Esther Greenglass

**Affiliations:** 1 163043 School of Educational Sciences and Psychology, University of Eastern Finland, Joensuu, Finland; 2 3835 Faculty of Behavioural Sciences, University of Helsinki, Helsinki, Finland; 3 7991 Faculty of Health, Department of Psychology, York University, Toronto, Canada

**Keywords:** anxiety, psychological distress, well-being, health psychology, depression, stress

## Abstract

**Background:** The Russian-Ukranian war (RUW) broke in 2022. Finland is a neighboring country of Russia. People in Finland could be assumed to be especially vulnerable to war-related stress. We examined the relationship between time spent following the RUW from media, sleep problems and psychological distress in university students. **Methods:** The participants were university students who responded anonymously to a questionnaire. They reported their age, gender, time spent following RUW, anxiety, depressive symptoms, and sleep problems. Statistical analyses were conducted using SPSS and Mplus for structural equation modeling. **Results:** The time spent following RUW from media was associated with greater psychological distress, and more sleep disturbances. Sleep disturbances accounted for more than 12% of the association between time spent following RUW and psychological distress. **Conclusions:** Present findings suggest that sleep problems should be taken into account when supporting students. Support programs should emphasize the importance of sleep in psychological well-being.

## Introduction

On February 24^th^ 2022, Russia launched an aggressive attack against Ukraine leading to widespread destruction and a humanitarian crisis. The conflict has resulted in extensive damage to people’s lives and infrastructure. The uncertainty and potential threat posed by the conflict have led to increased anxiety and mental health challenges. The constant flow of information has contributed to exposure to war-related stress among the population. Since Finland is a neighboring country of Russia, people in Finland could be assumed to be especially vulnerable to war-related stress.

With TV and a variety of social media available, it was possible to follow the war-related news and other information continuously. During COVID-19, there has been an increase in binge-watching, i.e. viewing online videos and streamed content any time of day and night which has been related to mental health problems and sleep problems (Alimoradi et al., 2022). Since spring 2022, it has been possible to follow the war in Ukraine from many different media. Pronounced exposure to news related to the war has been observed to be associated with higher fear concerning the future (Castro et al., 2023). Media-induced war trauma may impact many individuals who are not directly involved in the conflict, with the effects being intensified by the COVID-19 pandemic (Su et al., 2023). Watching the war may increase stress and the risk of mental illness which may also increase the risk of sleep problems.

Media exposure to disasters and large-scale violence can precipitate psychological distress such as anxiety and depression. Previous studies suggest that even without direct exposure to violence, perceived similarity of the viewer with the victims is associated with trauma and distress symptoms (Mash et al., 2016; Otto et al., 2007). Higher media exposure, in this case, the time spent following the Russian-Ukranian war (RUW), is assumed to be a significant source of stress. According to the Transactional Theory of Stress (Lazarus and Folkman, 1984), stressors are first evaluated in terms of their harmfulness and threat. If the stressor is perceived as threatening, the stressor is then secondarily appraised in terms of the individual’s resources to deal with the stressor. If coping resources are available, the stress of the experience can be alleviated. However, the war in Ukraine cannot be resolved with individual coping resources and an individual may feel threatened and helpless because of not being able to influence the situation or the suffering of people in Ukraine. Therefore, time spent following RUW may be especially harmful regarding a person’s psychological well-being. In addition, watching the war may increase the amount of negative emotions related to stress.

The war in Ukraine is a situation filled with worries and uncertainty for all people, including people living in Finland due to proximity to the war. Young adults, such as university students, are particularly vulnerable to psychological distress (Akram et al., 2020) as they also experience a high prevalence of stress due to several transitions in their lives: leaving home, moving to a new location, changes in peer groups and related changes in social relationships. Furthermore, studying at university is challenging in many ways, e.g. high degree of academic independence and expectations (Gardani et al., 2022). Given the challenging study environment, university students could be especially vulnerable to war-related stress.

In addition to stress, following the war can also result in intrusive and debilitating thoughts (Harrington and Cairney, 2021; Harrington et al., 2021). Intrusive thoughts occur when the ability to suppress unwanted thoughts is reduced and memory suppression has become deficient (Harrington and Cairney, 2021). It has been reported that impairment of top-down processes, i.e. use of general knowledge to understand and interpret sensory perceptions, may be related to the ability to suppress unwanted thoughts and emotions (Harrington and Cairney, 2021). Memory suppression is assumed to alleviate unpleasant experiences and to be related to healthy emotion regulation (Gagnepain et al., 2017). Poorer ability to suppress unwanted thoughts has been related to increased negative affect and may increase the risk of psychological problems and psychiatric disorders (Gagnepain et al., 2017).

Sleeping problems may diminish the ability to suppress intrusive thoughts through impairment of top-down processes (Harrington and Cairney, 2021). It has been reported that sleep-deprived persons are ineffective at suppressing intrusive thoughts (Harrington et al., 2021). Sleep deprivation can impair top-down inhibitory networks and increase intrusive thoughts that are related to emotion dysregulation (Harrington et al., 2021). The present research focuses on time spent following RUW, related stress, sleep problems, and psychological distress in university students. Findings from this study will add to the growing literature on watching the war and psychological distress.

The mechanisms linking stress with sleep problems can be physiological, psychological and behavioral in nature (Gardani et al., 2022). In the Spielman 3P Model, it is postulated that there are predisposing, precipitating and perpetuating factors that are involved in acute sleeping problems that become chronic (Spielman et al., 1987). In the present study, time spent watching the war in Ukraine is a perpetuating factor. First, time spent following RUW can reduce hours that a person sleeps. Time spent watching the war and related behavior may, in turn, enhance the risk of chronic sleep problems and psychological distress. A recent systematic review and meta-analysis among undergraduate students has shown a pooled association of 0.41 (12 studies) for stress and insomnia (Gardani et al., 2022).

In an explanatory model of sleep, the Psychobiological Inhibition Model, the four processes that facilitate good sleep are physiological and cognitive de-arousal, behavioral consolidation and emotional neutrality (Espie, 2023). Factors that are assumed to inhibit good sleep are failure of physiological and cognitive de-arousal, poor stimulus control in behavior, and emotional intensity and distress (Espie, 2023). This model is relevant to the present study because following the war may hinder downregulation of physiological arousal and lead to reduced mental alertness and more intrusive thoughts, i.e. failure of cognitive de-arousal. Physiological and cognitive arousal due to watching the war may increase difficulties falling asleep and lead to more negative emotions.

Theoretically, its assumed that there are three factors operating together in inducing sleep problems (Espie, 2023). The attention-intention-effort pathway proceeds so that stress induces difficulties with physiological de-arousal and attention to intrusive thoughts hinders cognitive de-arousal (Espie, 2023). Consequently, poor stimulus control may strengthen physiological, cognitive and emotional arousal, and make falling asleep difficult. These factors acting in concert can increase the risk of psychological distress. Recently, it has been reported that higher frequency of following RUW news and related higher use of social media were related to higher level of anxiety and depressive symptoms (Riad et al., 2022).

It has been shown that sleep is involved in emotion regulation (Tempesta et al., 2018). A study among university students has shown that sleep deprived subjects judged neutral stimuli more negatively than did well-rested subjects and increased negative mood (Tempesta et al., 2010). One effect of sleep has been related to enhancement of negative affective tone and subsequent assessment of stimuli as more negative (Tempesta et al., 2018). A vicious cycle of stress, sleep problems and intrusive thoughts related to emotion dysregulation, may be reinforcing and increase the risk of psychological distress and mental disorders.

The evidence about the relationship between time spent following the RUW, sleep problems and psychological distress in university students is still sparse. By examining these relationships, the study can provide valuable insights into the mental health needs of university students and contribute to the development of effective support systems.

**Figure 1.**
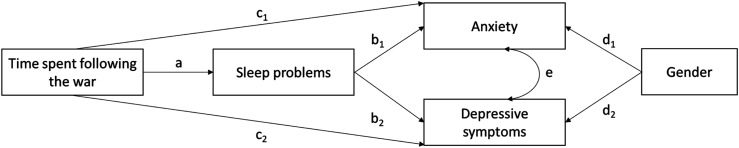
The tested model of the associations between time spent following RUW, sleep problems and psychological distress with standardized path coefficients. Path coefficients between gender and anxiety and gender and depressive symptoms indicate that men experience less anxiety and depressive symptoms compared to others. Sleep problems are controlled for age and gender (not shown). *p < .05, **p < .01, ***p < .00.

Our aim was to examine whether time spent following RUW is associated with psychological distress, defined as depression and anxiety, and whether sleep problems mediate this association. We hypothesize that more time spent following RUW would be associated with increased anxiety and depressive symptoms as well as more sleep problems. In addition, we hypothesize that these potential associations vary according to gender with women reporting more psychological distress (Greenglass et al., 2024). Furthermore, we hypothesize that time spent following RUW would be associated with anxiety and depressive symptoms partly through higher sleep problems. In other words, a partial mediation effect is predicted between time spent following RUW, sleep problems and distress. See Figure 1 for the hypothesized model.

## Methods

### Procedure

Data were collected as a part of a multi-national research collaboration (led by professor Esther Greenglass) on psychological factors and watching the war in Ukraine. A description of this project, the questionnaire and the data can be found on *The Open Science Framework.* The questionnaire was translated from English into Finnish and was posted online on the survey platform of *Webropol*. Data in Finland were collected from 25^th^ April until 20^th^ May 2022. The anonymous data used in the present study is stored confidentially and digitally by the research team. The University’s Ethics Committee provided approval for the conduct of this study and informed consent was obtained from participants before responding to the survey. No incentives were offered for participation.

### Participants

The data of the present cross-sectional study of university students were collected anonymously in the University of Eastern Finland. Data were cleaned according to several criteria. Participants who did not fill out the questionnaire were deleted. Those who took less than 3 minutes or more than 50 minutes to answer the questionnaire were dropped. Those who were not students and those who responded consecutively with the same response number more than 10 times were deleted. There were 233 eligible participants who were 29.5 years old (S.D. = 8.68) on average. Of the participants, 149 were female. Of the participants 8 (3%) reported gender as “other”. There were 45 (20.2%) first year students, 44 (19.7%) second year students, 40 (17.9%) third year students and 94 (42.2%) fourth year or more students. Study year was not linked to any of the relevant study variables, and thus, was dropped from further analyses.

### Measures

*Anxiety when following the war* was assessed with a modified version of the anxiety subscale of the Profile of Mood States (POMS; Shacham, 1983). It consists of 6 items with responses on a five-point scale ranging from 1 = *Not at all*, to 5 = *Extremely* (α = 0.88). Higher mean values indicate higher levels of anxiety.

*Depressive symptoms when following the war* were assessed with a modified version of the depression subscale of the Profile of Mood States (POMS) (Shacham, 1983). It consists of 8 items with responses on a five-point scale ranging from 1 = *Not at all,* to 5 = *Extremely* (α = 0.89). Higher values indicate more depressive symptoms. For both the depression and anxiety subscales, instructions were, “Indicate your feelings when you watch the war or read about it.”

*Time Watching the War* was measured with a single item developed by the authors to assess the amount of time spent following RUW. Participants responded with a number to the item, “In general, how many hours/per week do you spend watching or reading about the war on TV and/or the Internet, your phone, etc.?”

*Sleep problems* were assessed with the four-item Jenkins Sleep Problems Scale (Jenkins et al., 1988) that evaluates the frequency and intensity of sleep difficulties in respondents and was based on items that measured difficulty initiating sleep, waking up multiple times at night, early morning awakenings and non-restorative sleep which also correspond to the diagnostic symptoms for sleep disturbances. Participants were asked to rate on a scale of 1 (not at all) to 6 (every night) to what extent they had experienced these symptoms within the previous 4 weeks. We obtained a mean score for the items (Cronbach’s α = 0.72). The higher the value, the greater the level of sleep problems.

### Statistical analyses

Associations between the time spent following RUW, sleep problems, anxiety and depressive symptoms were examined with correlation coefficients and a theory-based structural equation model (SEM) regarding indirect associations. The model was tested with Mplus 8.8 (maximum likelihood estimator, 95% confidence interval with 5000 resamples for the indirect associations).

For the SEM, regarding fit indices, the comparative fit index (CFI) and Tucker–Lewis index (TLI) were considered to indicate satisfactory (≥.90) or good fit (≥.95), and both the root mean square error of approximation (RMSEA) and standardized root mean square residual (SRMR) indicated a satisfactory (≤.08) or good fit (≤.05; for the criteria, see (Little, 2013). The indirect association was considered statistically significant if the confidence interval of the product of the regression coefficients (ab_1_ for anxiety, ab_2_ for depressive symptoms) based on the 5000 resamples in the bootstrapped analysis did not include zero (see Figure 1). The strengths of the indirect associations are also illustrated as proportions of the total association between the focal variables (e.g., the proportion of the association between time spent following RUW and anxiety through sleep problems from the total association between time spent following RUW and anxiety).

In the hypothesized model, sleep problems were predicted by time spent following RUW. Anxiety and depressive symptoms were both predicted by time spent following RUW, sleep problems, and gender. Age and gender were controlled in the regression analysis because they have been shown to be related to sleep quality (Luca et al., 2015; Vitiello et al., 2004). Gender was included as two dummy variables (men/non-men and other/non-other).

Indirect associations between time spent following RUW and anxiety and depressive symptoms through sleep problems were examined in bootstrapped analyses by testing the confidence intervals of the products of appropriate regression path coefficients. The indirect association between time spent following RUW and depressive symptoms through sleep problems was tested by multiplying the regression coefficient between time and sleep problems (path a_1_) by the coefficient between sleep problems and depressive symptoms (path b_1_). In the same vein, the indirect association between time spent following RUW and anxiety through sleep problems was tested with the product of paths a_2_ and b_2_. See Figure 1 for the theoretical model.

## Results

The descriptive statistics of the sample and bivariate correlations between study variables are shown in Table 1. Older age was linked to higher amount of time spent following RUW. Time spent following RUW was linked to higher anxiety. Sleep problems were related to higher anxiety and higher depressive symptoms (Table 1).

**Table 1.** Descriptive statistics and bivariate correlations between study variables.

	Variable	Mean	S.D.	1	2	3	4	5	5a	5b	5c
1	Age	29.46	8.68								
2	Time spent following the war (hrs)	4.50	4.85	0.25***							
3	Anxiety	2.82	0.99	0.00	0.17**						
4	Depressive symptoms	2.51	0.94	0.04	0.11	0.74***					
5	Sleep problems	2.92	1.06	−0.01	0.11	0.25***	0.22***				
	5a Difficulties in falling asleep	1.63	1.37	−0.22***	0.07	0.26***	0.21***	0.64***			
	5b Waking up several times during the night	1.93	1.46	0.04	0.06	0.22***	0.14*	0.77***	0.33***		
	5c Difficulties in staying asleep	1.62	1.48	0.08	0.13*	0.16*	0.01	0.76***	0.31***	0.58***	
	5d felt tired after waking up	2.51	1.43	0.07	0.08	0.26***	0.27***	0.71***	0.33***	0.36***	0.36***

*p* < 0.05 **p < 0.01 ***p < 0.001; single items of the sleep problem measure have been indicated by 5a-5d.

Next, we examined the associations between time spent following RUW, anxiety and depressive symptoms, and indirect associations between them through sleep problems. The model regarding direct and indirect associations between the time spent following RUW, sleep problems, anxiety and depressive symptoms fit the data well (χ^2^ = 9.34, df = 6, p = .141, CFI = .984, TLI = .961, RMSEA = .051 [90% CI = .001; .108], SRMR = .032).

In the empirical model, the associations between time spent following RUW, sleep problems and psychological distress were tested with standardized path coefficients. Path coefficients between gender and anxiety, and between gender and depressive symptoms indicate that men experienced less anxiety and depressive symptoms than women.

**Figure 2.**
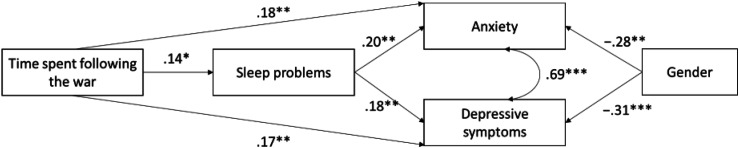
Theoretical model of the associations between time spent following RUW, sleep problems and psychological distress with standardized path coefficients.

The standardized regression paths and their statistical significance are shown in Figure 2.

In the empirical model, more time spent following RUW was associated with more sleep problems, greater anxiety and more depressive symptoms (Figure 2). Sleep problems were associated with more anxiety and depressive symptoms. Being male was associated with lower anxiety and lower depressive symptoms.

According to the bootstrap analyses, time spent following RUW also had an indirect association with anxiety through sleep problems (ab_1_ = 0.027, 95% CI = from 0.005 to 0.066). This indirect association accounted for 12.8% of the total association between time spent following RUW and anxiety.

Time spent following RUW had also an indirect association with depressive symptoms through sleep problems (ab_2_ = 0.025, 95% CI = from 0.006 to 0.061). This indirect association accounted for 12.8% of the total association between time spent following RUW and depressive symptoms.

## Discussion

We examined associations between time spent following RUW and psychological distress (i.e. anxiety and depressive symptoms). We examined also whether the association between time spent following RUW and psychological distress is mediated by sleep problems. We found that men experienced less anxiety and depressive symptoms than women. Sleep problems were associated with more anxiety and depressive symptoms. More time spent following RUW was associated with more sleep problems, anxiety and depressive symptoms. Sleep problems mediated the association between time spent following RUW and psychological distress.

Time spent following RUW was on average 4.5 hours per week. About one third of participants followed the war more than the average. This may have been due to the recency of the advent of the war (February, 2022), while data collection was in the spring of 2022. It has been reported that about 27.7% of Czech university students have checked the war news daily (Riad et al., 2022). We found that more time spent following RUW was related to greater anxiety, and that sleep problems were linked to psychological distress. RUW-related stress, the subsequent intrusive and debilitating thoughts, and negative feelings may worsen ability to sleep. A review of the literature has documented that insomnia and stress are interlinked and affect university students’ academic performance and mental health (Gardani et al., 2022).

The mechanisms linking stress with sleep problems can be physiological, such as a failure of normal de-arousal, psychological in the form of negative feelings and intrusive thoughts, and related behavioral compensation of napping or trying to sleep earlier than normal. War-related stress can increase sleep problems which can further impair the ability to cope with everyday stressors that may be social, interpersonal and academic in nature. Exposure to war-related information and related sleep problems together with impaired ability to cope with stress and feelings of anxiety may decrease psychological well-being.

The associations between war-related stress, sleep problems and psychological distress may be explained by the attention-intention-effort pathway which starts with difficulties in physiological de-arousal and proceeds to attention and intrusive thoughts that hinder cognitive de-arousal (Espie, 2023). Consequently, this process may strengthen physiological, cognitive and emotional arousal, and make falling asleep difficult. These factors acting together may also increase the risk of psychological distress.

According to the 3P model of insomnia (Spielman et al., 1987), time spent watching the war in Ukraine can be assumed to be a predisposing factor for sleep problems. Time spent watching the war and related behavior may increase the risk of chronic sleep problems and psychological distress. Sleep problems may be precipitating factors inthe association between war-related stress and psychological distress via physiological and psychological processes and related behavior. The present findings are in line with other research showing that time spent watching the RUW was associated with distress, particularly, depression (Begic et al., 2023).

The finding of a link between feelings of non-restorative sleep and depressive symptoms is in accordance with a previous finding reporting some differences in links between sleep problems and mental health (Becker et al., 2018). It has been shown that depressive symptoms (but not anxiety) are related to daytime dysfunction whereas anxiety (but not depressive symptoms) is linked to higher sleep disturbances (Becker et al., 2018). Sleep problems, especially feelings of non-restorative sleep, may have a significant impact on students’ daytime functioning in a form of poorer academic performance, emotion dysregulation and tiredness (Tempesta et al., 2018).

The association between higher RUW-related stress and higher psychological distress seems to be strengthened by sleep problems. It is also potential that sleep problems relate to increased perceptions of stress. In a study among general public during COVID-19 in China, it has been suggested that insomnia may contribute to higher perceived stress and may increase the risk of psychological distress (Mu et al., 2023). Our final model shows the associations between time spent following RUW, sleep problems, psychological distress and gender. We found that higher time spent following RUW was associated with more sleep problems, higher anxiety and higher depressive symptoms. Finns may be vulnerable to the RUW because their older generation participated in the 1939-1940 Winter War against the Soviet Union. War-related trauma can be transferred through generations (Castro-Vale et al., 2019; Leshem et al., 2025). This may have an impact on levels of anxiety and depression in today’s generation of Finns.

Time spent following RUW had an indirect association with anxiety and depressive symptoms through sleep problems. In other words, the association between time spent following RUW and psychological distress is partially mediated by sleep problems. These indirect associations accounted for 12.8% (anxiety) and 12.8% (depressive symptoms) of the total association between time spent following RUW and psychological distress.

The findings from our study can inform university policies and mental health services, ensuring they are better equipped to support students during times of global crisis. Developing research-based support systems can lead to improved academic performance and overall well-being among the student population.

### Limitations and strengths

There are some limitations which need to be considered when interpreting the present results. The study design is cross-sectional and therefore does not permit causal interpretations. Testing mediating associations in a cross-sectional study and related results can therefore be interpreted only from the statistical point of view. Even though it cannot be concluded that sleep problems cause a higher risk of psychological distress based on the present study, we suggest that it is worthwhile to take sleep problems into account in supporting university students to cope with RUW-related stress. The geographic proximity of Finland to Russia may have amplified the stress induced by following the RUW and therefore also influenced the present findings among Finnish university students. The indirect associations accounted for 12.8% of the total association between time spent following RUW and psychological distress which is quite modest in size. Furthermore, we suggest that the role of sleep problems should be examined in longitudinal research designs in future research. Finally, the study population is university students, and therefore the results cannot be directly generalized to other populations.

At the same time, there are several strengths in the present study. The data were collected in an optimal time window. The war started in February, 2022 and data collection in Finland took place from the 25^th^ April until 20^th^ May 2022. There was a 2-month period during which it was possible for students to follow the war from several sources of media before we began collecting data. The measures used in the present study have been used in other studies world-wide and therefore the results are comparable with international studies examining stress among university students. A recent systematic review and meta-analysis shows that there is a plethora of studies examining the associations between stress and sleep problems in students (Gardani et al., 2022). However, these reviewed studies include only a few studies from Europe (Gardani et al., 2022). Thus, our study produces scientific information about associations between time spent following RUW, sleep problems and psychological distress which is important and novel in a country with a shared border with Russia.

### Practical implications and suggestions for further research

Based on our findings, we suggest that it may be possible to support students when watching the war by developing counseling workshops, programs and strategies that decrease exposure to stressful news about the war and related stress and teach ways of effective coping with stress. It would be very important for students to recognize the potential risk of following war-related info and news for excessively long periods of time and also to identify how this may influence their sleep, emotions and psychological well-being. It is also important for students to seek help with their sleep problems in order to maintain their mental health.

Scientific information about the risks of war-related stress for psychological well-being is very important in order to maintain good sleep, optimal academic performance and necessary social ties with community. We suggest that the role of sleep problems in the associations between war-related stress and psychological well-being should be examined with longitudinal research designs in further studies. Psychological distress and stress appraisals occur when individuals perceive the environment as exceeding their resources (Lazarus and Folkman, 1984). From a theoretical perspective, stress appraisal can explain the relationship between a stressor (e.g. following the RUW) and negative psychological outcomes (e.g. anxiety and depressive symptoms).

## Conclusions

Following the RUW is stressful and associated with feelings of anxiety and depressive symptoms. Present findings emphasize the importance of taking sleep problems into account when supporting students and developing support programs designed to improve mental health. Supporting university students’ well-being should focus on alleviating stress caused by the unstable and distressing war situation and emphasizing the importance of adequate sleep and good sleep quality in psychological well-being.

## Data Availability

The dataset generated during and/or analyzed during the current study are available from the corresponding author on reasonable request. Ethics approval, participant permissions, and all other relevant approvals were granted for this data sharing. The data file from the current study are also available in the OSF repository at https://osf.io/whk48/.
